# 2-(1*H*-Benzoimidazol-2-yl)-6-ethoxy­phenol

**DOI:** 10.1107/S1600536809008071

**Published:** 2009-03-14

**Authors:** Chin Sing Yeap, Hadi Kargar, Reza Kia, Arezoo Jamshidvand, Hoong-Kun Fun

**Affiliations:** a X-ray Crystallography Unit, School of Physics, Universiti Sains Malaysia, 11800 USM, Penang, Malaysia; b Department of Chemistry, School of Science, Payame Noor University (PNU), Ardakan, Yazd, Iran

## Abstract

The title Schiff base compound, C_15_H_14_N_2_O_2_, consists of two crystallographically independent mol­ecules, *A* and *B*. Mol­ecule *A* is almost planar, whereas mol­ecule *B* is slightly twisted, the dihedral angles between the benzimidazole group and the benzene rings being 2.65 (12) and 13.17 (15)°, respectively. The methyl group of mol­ecule *B* is disordered over two positions, with a refined site-occupancy ratio of 0.581 (7):0.419 (7). In each mol­ecule, intra­molecular O—H⋯N hydrogen bonds generate *S*(6) ring motifs. In the crystal structure, both types of mol­ecules are linked *via* inter­molecular bifurcated N—H⋯O hydrogen bonds into one-dimensional extended chains along [010] and form *R*_1_^2^(5) ring motifs. The crystal structure is further stabilized by inter­molecular C—H⋯π and π–π inter­actions [centroid–centroid distances = 3.4758 (16)–3.596 (2) Å].

## Related literature

For hydrogen-bond motifs, see: Bernstein *et al.* (1995 ▶). For benzimidazole chemistry, reaction mechanisms and bioactivity, see, for example: Latif *et al.* (1983 ▶); Craigo *et al.* (1999 ▶); Gudmundsson *et al.* (2000 ▶); Trivedi *et al.*(2006 ▶); Kim *et al.* (1996 ▶); Ramla *et al.* (2006 ▶). For the stability of the temperature controller used in the data collection, see: Cosier & Glazer (1986 ▶).
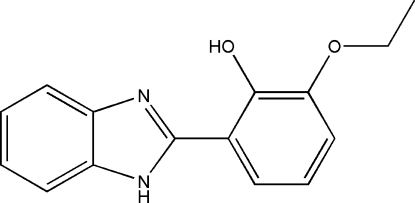


## Experimental

### 

#### Crystal data

C_15_H_14_N_2_O_2_
*M* *_r_* = 254.28
Monoclinic, 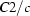

*a* = 22.5305 (4) Å
*b* = 12.0113 (2) Å
*c* = 21.4241 (3) Å
β = 120.449 (1)°
*V* = 4998.17 (14) Å^3^
*Z* = 16
Mo *K*α radiation
μ = 0.09 mm^−1^
*T* = 100 K
0.38 × 0.23 × 0.18 mm

#### Data collection

Bruker SMART APEXII CCD area-detector diffractometer
Absorption correction: multi-scan (**SADABS**; Bruker, 2005 ▶) *T*_min_ = 0.966, *T*_max_ = 0.984
49947 measured reflections
5171 independent reflections
4238 reflections with *I* > 2σ(*I*)
*R*_int_ = 0.043

#### Refinement

*R*[*F* ^2^ > 2σ(*F* ^2^)] = 0.067
*wR*(*F* ^2^) = 0.151
*S* = 1.19
5171 reflections
372 parameters
1 restraint
H atoms treated by a mixture of independent and constrained refinement
Δρ_max_ = 0.57 e Å^−3^
Δρ_min_ = −0.48 e Å^−3^

### 

Data collection: *APEX2* (Bruker, 2005 ▶); cell refinement: *SAINT* (Bruker, 2005 ▶); data reduction: *SAINT*; program(s) used to solve structure: *SHELXTL* (Sheldrick, 2008 ▶); program(s) used to refine structure: *SHELXTL*; molecular graphics: *SHELXTL*; software used to prepare material for publication: *SHELXTL* and *PLATON* (Spek, 2009 ▶).

## Supplementary Material

Crystal structure: contains datablocks global, I. DOI: 10.1107/S1600536809008071/lh2781sup1.cif

Structure factors: contains datablocks I. DOI: 10.1107/S1600536809008071/lh2781Isup2.hkl

Additional supplementary materials:  crystallographic information; 3D view; checkCIF report

## Figures and Tables

**Table 1:** Hydrogen-bond geometry (Å, °)

*D*—H⋯*A*	*D*—H	H⋯*A*	*D*⋯*A*	*D*—H⋯*A*
O1*A*—H1*OA*⋯N1*A*	0.91 (4)	1.67 (4)	2.557 (4)	164 (4)
N2*A*—H1*NA*⋯O1*B*	0.81 (4)	2.14 (4)	2.865 (3)	149 (3)
N2*A*—H1*NA*⋯O2*B*	0.81 (4)	2.57 (4)	3.199 (3)	136 (3)
O1*B*—H1*OB*⋯N1*B*	0.97 (4)	1.67 (4)	2.567 (3)	151 (3)
N2*B*—H1*NB*⋯O1*A*^i^	0.94 (4)	1.95 (4)	2.877 (3)	167 (4)
N2*B*—H1*NB*⋯O2*A*^i^	0.94 (4)	2.55 (4)	3.136 (3)	121 (3)
C4*A*—H4*AA*⋯*Cg*1^ii^	0.93	2.80	3.590 (4)	143
C14*B*—H14*C*⋯*Cg*2^iii^	0.97	2.84	3.721 (5)	152
C15*B*—H15*D*⋯*Cg*3^iv^	0.96	2.76	3.715 (8)	176

Symmetry codes: (i) 
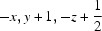
; (ii) 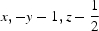; (iii) 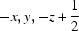; (iv) 
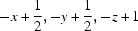
. *Cg*1, *Cg*2 and *Cg*3 are the centroids of the C8*B*–C13*B*, C1*B*–C6*B* and N1*A*–C1*A*–C6*A*–N2*A*–C7*A* rings, respectively.

**Fig. 1.**
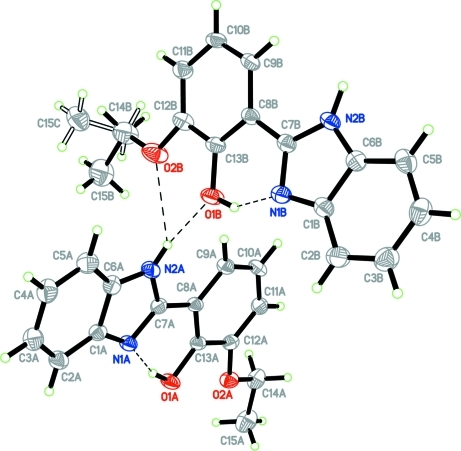
The molecular structure of the title compound with atom labels and 50% probability ellipsoids for non-H atoms. Intra- and intermolecular hydrogen bonds are shown as dashed lines. The open bond indicates the minor component of disorder.

**Fig. 2.**
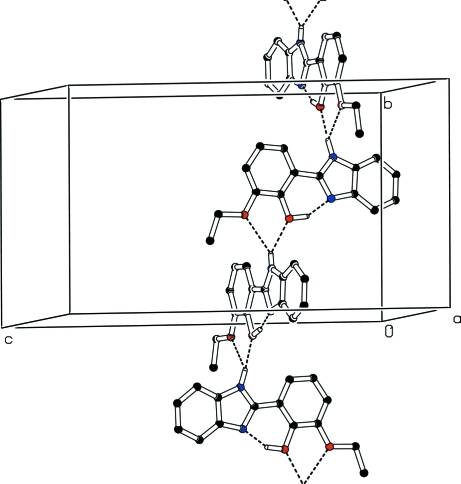
Part of the crystal structure of the title compound with hydrogen bonds shown as dashed lines. The disorder is not shown and only H atoms involved in hydrogen bonds are drawn.

## supplementary crystallographic information

### Comment

Benzimidazoles are used widely in biological applications and as pharmaceutical agents (Craigo *et al.*, 1999; Gudmundsson *et al.*, 2000; Trivedi *et al.*, 2006). They are also used as topoisomerase I inhibitors (Kim *et al.*, 1996) and for antitumor activity (Ramla *et al.*, 2006). Due to these important applications, many synthetic routes towards benzimidazoles have been developed. They can, for example, be synthesized by the reaction of phenolic aldehydes with *o*-phenylenediamine (Latif *et al.,*, 1983). Based on this route the title compound was synthesized and its crystal structure is reported here.

The asymmetric unit of the title compound, Fig. 1, consists of two crystallographically independent molecules, *A* and *B* with a slightly different conformations due to a disordered group. Intramolecular O—H···N hydrogen bonds generate *S*(6) ring motifs (Bernstein *et al.,* 1995). The two molecules *A* and *B* are linked together by a bifurcated hydrogen bond involving the two oxygen atoms of the hydroxy and ethoxy groups with a *R*_1_^2^(5) ring motif. The molecule *A* is almost planar wheares the molecule *B* is slightly twisted with the dihedral angles between the benzimidazole and the phenyl rings being 2.65 (12) and 13.17 (15) °, respectively. The methyl group of molecule *B* is disordered over two positions with a refined site-occupancy ratio of 0.581 (7):0.419 (7). The crystal structure is further stabilized by intermolecular C—H···π [*Cg*1, *Cg*2 and *Cg*3 are the centroids of the C8B–C13B, C1B–C6B and N1A/C1A/C6A/N2A/C7A rings] (Table 1) and π-π interactions [*Cg*1···*Cg*4^iii^ = 3.596 (2) and *Cg*3···*Cg*5^iii^ = 3.4758 (16) Å; (iii) -x, y, 1/2 - z ; *Cg*4 and *Cg*5 are the centroids of the N1B/C1B/C6B/N2B/C7B and C8A–C13A rings]. In the crystal structure, molecules are linked together into 1-D extended chains along the [0 1 0] direction (Fig. 2).

### Experimental

An ethanolic solution (50 ml) of 3-ethoxy-salicylaldehyde (2 mmol, 332 mg) was added to 1,2-phenylenediamine (1 mmol, 217 mg). The mixture was refluxed for 2 h, and cooled to room temperature. The resulting colourless powder was filtered, washed with cooled ethanol and dried in *vacuo*. Single crystals suitable for *X*-ray diffraction were obtained from an methanol solution at room temperature.

### Refinement

O1A, O1B and N-bound hydrogen atoms were located from the diffrence Fourier map and refined freely. The rest of the hydrogen atoms were positioned geometrically with a riding model approximation with C—H = 0.93-0.97 Å and U_iso_(H) = 1.2 or 1.5 (C & O). A rotating group model was used for methyl group.

### Crystal data

**Table:**

C_15_H_14_N_2_O_2_	*F*(000) = 2144
*M**_r_* = 254.28	*D*_x_ = 1.352 Mg m^−^^3^
Monoclinic, *C*2/*c*	Mo *K*α radiation, λ = 0.71073 Å
Hall symbol: -C 2yc	Cell parameters from 9961 reflections
*a* = 22.5305 (4) Å	θ = 2.5–32.2°
*b* = 12.0113 (2) Å	µ = 0.09 mm^−^^1^
*c* = 21.4241 (3) Å	*T* = 100 K
β = 120.449 (1)°	Block, yellow
*V* = 4998.17 (14) Å^3^	0.38 × 0.23 × 0.18 mm
*Z* = 16	

### Data collection

**Table:**

Bruker SMART APEXII CCD area-detector diffractometer	5171 independent reflections
Radiation source: fine-focus sealed tube	4238 reflections with *I* > 2σ(*I*)
graphite	*R*_int_ = 0.043
φ and ω scans	θ_max_ = 26.5°, θ_min_ = 2.0°
Absorption correction: multi-scan (*SADABS*; Bruker, 2005)	*h* = −28→28
*T*_min_ = 0.966, *T*_max_ = 0.984	*k* = −14→15
49947 measured reflections	*l* = −26→26

### Refinement

**Table:**

Refinement on *F*^2^	Primary atom site location: structure-invariant direct methods
Least-squares matrix: full	Secondary atom site location: difference Fourier map
*R*[*F*^2^ > 2σ(*F*^2^)] = 0.067	Hydrogen site location: inferred from neighbouring sites
*wR*(*F*^2^) = 0.151	H atoms treated by a mixture of independent and constrained refinement
*S* = 1.19	*w* = 1/[σ^2^(*F*_o_^2^) + (0.022*P*)^2^ + 16.3221*P*] where *P* = (*F*_o_^2^ + 2*F*_c_^2^)/3
5171 reflections	(Δ/σ)_max_ < 0.001
372 parameters	Δρ_max_ = 0.57 e Å^−^^3^
1 restraint	Δρ_min_ = −0.48 e Å^−^^3^

### Special details

**Table:**

Experimental. The crystal was placed in the cold stream of an Oxford Cyrosystems Cobra open-flow nitrogen cryostat (Cosier & Glazer, 1986) operating at 100.0 (1) K.
Geometry. All esds (except the esd in the dihedral angle between two l.s. planes) are estimated using the full covariance matrix. The cell esds are taken into account individually in the estimation of esds in distances, angles and torsion angles; correlations between esds in cell parameters are only used when they are defined by crystal symmetry. An approximate (isotropic) treatment of cell esds is used for estimating esds involving l.s. planes.
Refinement. Refinement of F^2^ against ALL reflections. The weighted R-factor wR and goodness of fit S are based on F^2^, conventional R-factors R are based on F, with F set to zero for negative F^2^. The threshold expression of F^2^ > 2sigma(F^2^) is used only for calculating R-factors(gt) etc. and is not relevant to the choice of reflections for refinement. R-factors based on F^2^ are statistically about twice as large as those based on F, and R- factors based on ALL data will be even larger.

### Fractional atomic coordinates and isotropic or equivalent isotropic displacement parameters (Å^2^)

**Table:**

	*x*	*y*	*z*	*U*_iso_*/*U*_eq_	Occ. (<1)
O1A	0.04313 (11)	−0.05359 (15)	0.34802 (11)	0.0343 (5)	
O2A	−0.04669 (10)	−0.05371 (16)	0.38878 (11)	0.0352 (5)	
N1A	0.12309 (11)	0.04703 (18)	0.31444 (12)	0.0278 (5)	
N2A	0.12918 (13)	0.2321 (2)	0.31223 (14)	0.0328 (5)	
C1A	0.16902 (14)	0.0757 (2)	0.29195 (14)	0.0280 (6)	
C2A	0.20683 (15)	0.0094 (2)	0.27078 (16)	0.0345 (6)	
H2AA	0.2047	−0.0678	0.2714	0.041*	
C3A	0.24743 (16)	0.0632 (3)	0.24891 (17)	0.0389 (7)	
H3AA	0.2731	0.0209	0.2346	0.047*	
C4A	0.25106 (16)	0.1776 (3)	0.24763 (18)	0.0413 (7)	
H4AA	0.2792	0.2104	0.2326	0.050*	
C5A	0.21403 (16)	0.2450 (3)	0.26805 (17)	0.0408 (7)	
H5AA	0.2165	0.3222	0.2670	0.049*	
C6A	0.17291 (14)	0.1923 (2)	0.29023 (15)	0.0318 (6)	
C7A	0.09976 (13)	0.1427 (2)	0.32507 (13)	0.0267 (5)	
C8A	0.04901 (13)	0.1485 (2)	0.34835 (14)	0.0257 (5)	
C9A	0.02514 (14)	0.2496 (2)	0.36000 (15)	0.0319 (6)	
H9AA	0.0424	0.3165	0.3540	0.038*	
C10A	−0.02370 (15)	0.2501 (2)	0.38028 (16)	0.0362 (7)	
H10A	−0.0398	0.3175	0.3873	0.043*	
C11A	−0.04945 (14)	0.1499 (2)	0.39045 (15)	0.0328 (6)	
H11A	−0.0824	0.1511	0.4043	0.039*	
C12A	−0.02616 (14)	0.0499 (2)	0.38002 (14)	0.0290 (6)	
C13A	0.02312 (13)	0.0480 (2)	0.35843 (13)	0.0255 (5)	
C14A	−0.09118 (15)	−0.0595 (3)	0.41821 (17)	0.0405 (7)	
H14A	−0.1346	−0.0230	0.3863	0.049*	
H14B	−0.0700	−0.0237	0.4652	0.049*	
C15A	−0.10244 (18)	−0.1825 (3)	0.4250 (2)	0.0546 (9)	
H15A	−0.1308	−0.1911	0.4463	0.082*	
H15B	−0.0588	−0.2181	0.4553	0.082*	
H15C	−0.1248	−0.2163	0.3779	0.082*	
O1B	0.08605 (12)	0.45323 (16)	0.25795 (12)	0.0409 (5)	
O2B	0.17852 (13)	0.46703 (18)	0.39287 (12)	0.0530 (6)	
N1B	0.00726 (12)	0.54159 (19)	0.13347 (13)	0.0332 (5)	
N2B	−0.01822 (12)	0.72236 (19)	0.12353 (13)	0.0303 (5)	
C1B	−0.04540 (15)	0.5626 (2)	0.06352 (16)	0.0335 (6)	
C2B	−0.08028 (17)	0.4918 (3)	0.00407 (17)	0.0438 (8)	
H2BA	−0.0692	0.4166	0.0075	0.053*	
C3B	−0.13164 (17)	0.5368 (3)	−0.06005 (17)	0.0461 (8)	
H3BA	−0.1548	0.4912	−0.1005	0.055*	
C4B	−0.14955 (16)	0.6492 (3)	−0.06553 (17)	0.0448 (8)	
H4BA	−0.1852	0.6762	−0.1092	0.054*	
C5B	−0.11554 (16)	0.7216 (3)	−0.00752 (17)	0.0393 (7)	
H5BA	−0.1274	0.7965	−0.0112	0.047*	
C6B	−0.06268 (14)	0.6763 (2)	0.05654 (15)	0.0313 (6)	
C7B	0.02231 (14)	0.6394 (2)	0.16742 (15)	0.0295 (6)	
C8B	0.07626 (14)	0.6529 (2)	0.24254 (15)	0.0286 (6)	
C9B	0.10079 (14)	0.7581 (2)	0.27454 (16)	0.0303 (6)	
H9BA	0.0819	0.8224	0.2475	0.036*	
C10B	0.15256 (15)	0.7658 (2)	0.34562 (16)	0.0332 (6)	
H10B	0.1688	0.8356	0.3660	0.040*	
C11B	0.18127 (16)	0.6703 (2)	0.38783 (16)	0.0357 (7)	
H11B	0.2165	0.6764	0.4358	0.043*	
C12B	0.15684 (16)	0.5670 (2)	0.35757 (16)	0.0368 (7)	
C13B	0.10542 (15)	0.5578 (2)	0.28474 (16)	0.0319 (6)	
C14B	0.2276 (2)	0.4683 (3)	0.46948 (18)	0.0576 (10)	
H14C	0.2090	0.5088	0.4949	0.069*	0.581 (7)
H14D	0.2698	0.5046	0.4788	0.069*	0.581 (7)
H14E	0.2218	0.4028	0.4915	0.069*	0.419 (7)
H14F	0.2208	0.5323	0.4920	0.069*	0.419 (7)
C15B	0.2415 (4)	0.3527 (6)	0.4943 (4)	0.070 (2)	0.581 (7)
H15D	0.2734	0.3510	0.5456	0.104*	0.581 (7)
H15E	0.2609	0.3139	0.4697	0.104*	0.581 (7)
H15F	0.1993	0.3172	0.4841	0.104*	0.581 (7)
C15C	0.2967 (3)	0.4728 (8)	0.4792 (5)	0.063 (3)	0.419 (7)
H15G	0.3303	0.4739	0.5300	0.095*	0.419 (7)
H15H	0.3012	0.5390	0.4568	0.095*	0.419 (7)
H15I	0.3039	0.4085	0.4573	0.095*	0.419 (7)
H1OA	0.0731 (18)	−0.030 (3)	0.3339 (18)	0.052 (10)*	
H1NA	0.1191 (17)	0.297 (3)	0.3128 (18)	0.048 (10)*	
H1OB	0.0555 (19)	0.462 (3)	0.206 (2)	0.061 (11)*	
H1NB	−0.0186 (17)	0.797 (3)	0.1365 (18)	0.052 (10)*	

### Atomic displacement parameters (Å^2^)

**Table:**

	*U*^11^	*U*^22^	*U*^33^	*U*^12^	*U*^13^	*U*^23^
O1A	0.0431 (12)	0.0193 (9)	0.0518 (13)	−0.0025 (8)	0.0323 (11)	−0.0009 (8)
O2A	0.0383 (11)	0.0298 (10)	0.0448 (12)	−0.0082 (9)	0.0264 (10)	−0.0019 (9)
N1A	0.0325 (12)	0.0206 (11)	0.0335 (12)	−0.0002 (9)	0.0191 (10)	−0.0002 (9)
N2A	0.0359 (13)	0.0209 (12)	0.0431 (14)	0.0007 (10)	0.0210 (12)	0.0060 (10)
C1A	0.0303 (14)	0.0240 (13)	0.0278 (13)	−0.0017 (11)	0.0133 (11)	0.0029 (10)
C2A	0.0376 (16)	0.0281 (14)	0.0389 (16)	0.0001 (12)	0.0202 (13)	0.0017 (12)
C3A	0.0388 (16)	0.0428 (17)	0.0399 (16)	0.0019 (14)	0.0236 (14)	0.0032 (13)
C4A	0.0374 (16)	0.0426 (18)	0.0490 (18)	−0.0001 (14)	0.0256 (14)	0.0123 (14)
C5A	0.0425 (17)	0.0315 (16)	0.0520 (19)	−0.0031 (13)	0.0265 (15)	0.0102 (14)
C6A	0.0302 (14)	0.0306 (15)	0.0331 (15)	0.0008 (11)	0.0149 (12)	0.0082 (12)
C7A	0.0263 (13)	0.0243 (13)	0.0247 (13)	−0.0020 (10)	0.0092 (11)	0.0019 (10)
C8A	0.0277 (13)	0.0202 (12)	0.0259 (13)	−0.0006 (10)	0.0111 (11)	0.0019 (10)
C9A	0.0359 (15)	0.0231 (13)	0.0357 (15)	0.0007 (11)	0.0174 (13)	0.0030 (11)
C10A	0.0429 (17)	0.0242 (14)	0.0427 (17)	0.0071 (12)	0.0226 (14)	−0.0007 (12)
C11A	0.0321 (14)	0.0343 (15)	0.0339 (15)	0.0000 (12)	0.0182 (13)	−0.0027 (12)
C12A	0.0287 (14)	0.0277 (14)	0.0273 (14)	−0.0035 (11)	0.0117 (11)	0.0006 (11)
C13A	0.0288 (13)	0.0202 (12)	0.0246 (13)	−0.0017 (10)	0.0115 (11)	0.0000 (10)
C14A	0.0353 (16)	0.0524 (19)	0.0362 (16)	−0.0049 (14)	0.0199 (14)	0.0054 (14)
C15A	0.049 (2)	0.059 (2)	0.059 (2)	−0.0151 (17)	0.0297 (18)	0.0116 (18)
O1B	0.0528 (13)	0.0209 (10)	0.0370 (12)	0.0008 (9)	0.0139 (10)	−0.0005 (8)
O2B	0.0737 (17)	0.0314 (12)	0.0355 (12)	0.0072 (11)	0.0143 (12)	0.0051 (9)
N1B	0.0361 (13)	0.0250 (12)	0.0356 (13)	0.0000 (10)	0.0159 (11)	−0.0009 (10)
N2B	0.0370 (13)	0.0203 (11)	0.0390 (13)	0.0024 (10)	0.0233 (11)	0.0035 (10)
C1B	0.0365 (15)	0.0277 (14)	0.0379 (16)	0.0032 (12)	0.0200 (13)	0.0030 (12)
C2B	0.0485 (19)	0.0346 (17)	0.0410 (17)	−0.0007 (14)	0.0172 (15)	−0.0021 (13)
C3B	0.0463 (18)	0.0474 (19)	0.0372 (17)	−0.0037 (15)	0.0158 (15)	−0.0010 (14)
C4B	0.0397 (17)	0.055 (2)	0.0370 (17)	0.0017 (15)	0.0172 (14)	0.0107 (15)
C5B	0.0436 (17)	0.0351 (16)	0.0449 (18)	0.0063 (13)	0.0265 (15)	0.0145 (13)
C6B	0.0328 (14)	0.0309 (14)	0.0370 (15)	−0.0007 (12)	0.0228 (13)	0.0043 (12)
C7B	0.0326 (14)	0.0255 (14)	0.0380 (15)	0.0012 (11)	0.0234 (13)	0.0011 (11)
C8B	0.0314 (14)	0.0243 (13)	0.0384 (15)	0.0001 (11)	0.0237 (12)	−0.0023 (11)
C9B	0.0370 (15)	0.0202 (13)	0.0438 (16)	0.0021 (11)	0.0278 (14)	0.0009 (11)
C10B	0.0397 (16)	0.0238 (14)	0.0452 (17)	−0.0052 (12)	0.0282 (14)	−0.0108 (12)
C11B	0.0402 (16)	0.0329 (15)	0.0348 (15)	0.0002 (12)	0.0196 (13)	−0.0061 (12)
C12B	0.0462 (17)	0.0288 (15)	0.0373 (16)	0.0029 (13)	0.0225 (14)	−0.0005 (12)
C13B	0.0383 (15)	0.0231 (13)	0.0390 (16)	−0.0022 (11)	0.0230 (13)	−0.0051 (11)
C14B	0.071 (3)	0.046 (2)	0.0390 (19)	0.0051 (18)	0.0161 (18)	0.0034 (16)
C15B	0.067 (5)	0.070 (5)	0.044 (4)	0.014 (4)	0.008 (3)	0.013 (3)
C15C	0.062 (6)	0.049 (5)	0.068 (6)	0.014 (4)	0.024 (5)	0.009 (4)

### Geometric parameters (Å, °)

**Table:**

O1A—C13A	1.358 (3)	N1B—C7B	1.332 (3)
O1A—H1OA	0.91 (4)	N1B—C1B	1.384 (4)
O2A—C12A	1.374 (3)	N2B—C7B	1.356 (3)
O2A—C14A	1.431 (3)	N2B—C6B	1.383 (4)
N1A—C7A	1.330 (3)	N2B—H1NB	0.94 (4)
N1A—C1A	1.388 (3)	C1B—C2B	1.396 (4)
N2A—C7A	1.361 (3)	C1B—C6B	1.407 (4)
N2A—C6A	1.376 (4)	C2B—C3B	1.381 (4)
N2A—H1NA	0.82 (4)	C2B—H2BA	0.9300
C1A—C2A	1.399 (4)	C3B—C4B	1.397 (5)
C1A—C6A	1.404 (4)	C3B—H3BA	0.9300
C2A—C3A	1.381 (4)	C4B—C5B	1.387 (5)
C2A—H2AA	0.9300	C4B—H4BA	0.9300
C3A—C4A	1.378 (4)	C5B—C6B	1.393 (4)
C3A—H3AA	0.9300	C5B—H5BA	0.9300
C4A—C5A	1.383 (4)	C7B—C8B	1.453 (4)
C4A—H4AA	0.9300	C8B—C13B	1.396 (4)
C5A—C6A	1.390 (4)	C8B—C9B	1.410 (4)
C5A—H5AA	0.9300	C9B—C10B	1.374 (4)
C7A—C8A	1.463 (4)	C9B—H9BA	0.9300
C8A—C9A	1.400 (4)	C10B—C11B	1.400 (4)
C8A—C13A	1.404 (3)	C10B—H10B	0.9300
C9A—C10A	1.373 (4)	C11B—C12B	1.378 (4)
C9A—H9AA	0.9300	C11B—H11B	0.9300
C10A—C11A	1.400 (4)	C12B—C13B	1.399 (4)
C10A—H10A	0.9300	C14B—C15C	1.4633 (10)
C11A—C12A	1.373 (4)	C14B—C15B	1.464 (7)
C11A—H11A	0.9300	C14B—H14C	0.9700
C12A—C13A	1.403 (4)	C14B—H14D	0.9700
C14A—C15A	1.519 (5)	C14B—H14E	0.9599
C14A—H14A	0.9700	C14B—H14F	0.9600
C14A—H14B	0.9700	C15B—H14E	0.7332
C15A—H15A	0.9600	C15B—H15D	0.9600
C15A—H15B	0.9600	C15B—H15E	0.9600
C15A—H15C	0.9600	C15B—H15F	0.9600
O1B—C13B	1.358 (3)	C15C—H15G	0.9600
O1B—H1OB	0.97 (4)	C15C—H15H	0.9600
O2B—C12B	1.370 (4)	C15C—H15I	0.9600
O2B—C14B	1.441 (4)		
			
C13A—O1A—H1OA	98 (2)	C3B—C2B—C1B	118.0 (3)
C12A—O2A—C14A	117.6 (2)	C3B—C2B—H2BA	121.0
C7A—N1A—C1A	105.9 (2)	C1B—C2B—H2BA	121.0
C7A—N2A—C6A	107.6 (2)	C2B—C3B—C4B	121.5 (3)
C7A—N2A—H1NA	126 (2)	C2B—C3B—H3BA	119.3
C6A—N2A—H1NA	126 (2)	C4B—C3B—H3BA	119.3
N1A—C1A—C2A	130.9 (2)	C5B—C4B—C3B	121.7 (3)
N1A—C1A—C6A	108.9 (2)	C5B—C4B—H4BA	119.1
C2A—C1A—C6A	120.2 (3)	C3B—C4B—H4BA	119.1
C3A—C2A—C1A	117.5 (3)	C4B—C5B—C6B	116.5 (3)
C3A—C2A—H2AA	121.3	C4B—C5B—H5BA	121.8
C1A—C2A—H2AA	121.3	C6B—C5B—H5BA	121.8
C4A—C3A—C2A	121.9 (3)	N2B—C6B—C5B	132.4 (3)
C4A—C3A—H3AA	119.0	N2B—C6B—C1B	105.2 (2)
C2A—C3A—H3AA	119.0	C5B—C6B—C1B	122.4 (3)
C3A—C4A—C5A	121.7 (3)	N1B—C7B—N2B	111.9 (2)
C3A—C4A—H4AA	119.1	N1B—C7B—C8B	122.8 (2)
C5A—C4A—H4AA	119.1	N2B—C7B—C8B	125.3 (2)
C4A—C5A—C6A	117.1 (3)	C13B—C8B—C9B	118.6 (3)
C4A—C5A—H5AA	121.5	C13B—C8B—C7B	118.8 (2)
C6A—C5A—H5AA	121.5	C9B—C8B—C7B	122.7 (2)
N2A—C6A—C5A	132.5 (3)	C10B—C9B—C8B	120.2 (3)
N2A—C6A—C1A	105.8 (2)	C10B—C9B—H9BA	119.9
C5A—C6A—C1A	121.6 (3)	C8B—C9B—H9BA	119.9
N1A—C7A—N2A	111.8 (2)	C9B—C10B—C11B	121.0 (3)
N1A—C7A—C8A	123.0 (2)	C9B—C10B—H10B	119.5
N2A—C7A—C8A	125.2 (2)	C11B—C10B—H10B	119.5
C9A—C8A—C13A	119.4 (2)	C12B—C11B—C10B	119.3 (3)
C9A—C8A—C7A	122.6 (2)	C12B—C11B—H11B	120.3
C13A—C8A—C7A	118.0 (2)	C10B—C11B—H11B	120.3
C10A—C9A—C8A	120.2 (3)	O2B—C12B—C11B	125.7 (3)
C10A—C9A—H9AA	119.9	O2B—C12B—C13B	114.1 (3)
C8A—C9A—H9AA	119.9	C11B—C12B—C13B	120.2 (3)
C9A—C10A—C11A	120.5 (3)	O1B—C13B—C8B	122.5 (3)
C9A—C10A—H10A	119.8	O1B—C13B—C12B	116.8 (3)
C11A—C10A—H10A	119.8	C8B—C13B—C12B	120.6 (3)
C12A—C11A—C10A	120.2 (3)	O2B—C14B—C15C	108.0 (5)
C12A—C11A—H11A	119.9	O2B—C14B—C15B	107.6 (4)
C10A—C11A—H11A	119.9	C15C—C14B—C15B	88.1 (5)
C11A—C12A—O2A	126.0 (2)	O2B—C14B—H14C	110.2
C11A—C12A—C13A	120.0 (2)	C15C—C14B—H14C	129.3
O2A—C12A—C13A	114.1 (2)	C15B—C14B—H14C	110.2
O1A—C13A—C12A	117.0 (2)	O2B—C14B—H14D	110.2
O1A—C13A—C8A	123.3 (2)	C15B—C14B—H14D	110.2
C12A—C13A—C8A	119.8 (2)	H14C—C14B—H14D	108.5
O2A—C14A—C15A	106.2 (3)	O2B—C14B—H14E	109.5
O2A—C14A—H14A	110.5	C15C—C14B—H14E	111.1
C15A—C14A—H14A	110.5	H14C—C14B—H14E	85.9
O2A—C14A—H14B	110.5	H14D—C14B—H14E	129.1
C15A—C14A—H14B	110.5	O2B—C14B—H14F	110.8
H14A—C14A—H14B	108.7	C15C—C14B—H14F	109.1
C14A—C15A—H15A	109.5	C15B—C14B—H14F	129.5
C14A—C15A—H15B	109.5	H14D—C14B—H14F	85.8
H15A—C15A—H15B	109.5	H14E—C14B—H14F	108.3
C14A—C15A—H15C	109.5	C14B—C15B—H15D	109.5
H15A—C15A—H15C	109.5	H14E—C15B—H15D	100.5
H15B—C15A—H15C	109.5	C14B—C15B—H15E	109.5
C13B—O1B—H1OB	106 (2)	H14E—C15B—H15E	141.2
C12B—O2B—C14B	118.2 (3)	C14B—C15B—H15F	109.5
C7B—N1B—C1B	105.8 (2)	H14E—C15B—H15F	81.8
C7B—N2B—C6B	107.8 (2)	C14B—C15C—H15G	109.5
C7B—N2B—H1NB	127 (2)	C14B—C15C—H15H	109.5
C6B—N2B—H1NB	125 (2)	H15G—C15C—H15H	109.5
N1B—C1B—C2B	130.9 (3)	C14B—C15C—H15I	109.5
N1B—C1B—C6B	109.4 (3)	H15G—C15C—H15I	109.5
C2B—C1B—C6B	119.8 (3)	H15H—C15C—H15I	109.5
			
C7A—N1A—C1A—C2A	−177.4 (3)	C7B—N1B—C1B—C6B	−1.1 (3)
C7A—N1A—C1A—C6A	0.6 (3)	N1B—C1B—C2B—C3B	179.8 (3)
N1A—C1A—C2A—C3A	178.1 (3)	C6B—C1B—C2B—C3B	−0.6 (5)
C6A—C1A—C2A—C3A	0.3 (4)	C1B—C2B—C3B—C4B	−1.2 (5)
C1A—C2A—C3A—C4A	−0.1 (5)	C2B—C3B—C4B—C5B	1.7 (5)
C2A—C3A—C4A—C5A	−0.2 (5)	C3B—C4B—C5B—C6B	−0.1 (5)
C3A—C4A—C5A—C6A	0.2 (5)	C7B—N2B—C6B—C5B	178.4 (3)
C7A—N2A—C6A—C5A	177.6 (3)	C7B—N2B—C6B—C1B	−0.6 (3)
C7A—N2A—C6A—C1A	−1.1 (3)	C4B—C5B—C6B—N2B	179.3 (3)
C4A—C5A—C6A—N2A	−178.5 (3)	C4B—C5B—C6B—C1B	−1.8 (4)
C4A—C5A—C6A—C1A	0.0 (4)	N1B—C1B—C6B—N2B	1.1 (3)
N1A—C1A—C6A—N2A	0.3 (3)	C2B—C1B—C6B—N2B	−178.6 (3)
C2A—C1A—C6A—N2A	178.6 (2)	N1B—C1B—C6B—C5B	−178.1 (2)
N1A—C1A—C6A—C5A	−178.6 (3)	C2B—C1B—C6B—C5B	2.2 (4)
C2A—C1A—C6A—C5A	−0.3 (4)	C1B—N1B—C7B—N2B	0.7 (3)
C1A—N1A—C7A—N2A	−1.3 (3)	C1B—N1B—C7B—C8B	−179.1 (2)
C1A—N1A—C7A—C8A	179.1 (2)	C6B—N2B—C7B—N1B	0.0 (3)
C6A—N2A—C7A—N1A	1.5 (3)	C6B—N2B—C7B—C8B	179.7 (2)
C6A—N2A—C7A—C8A	−178.9 (2)	N1B—C7B—C8B—C13B	−11.6 (4)
N1A—C7A—C8A—C9A	179.5 (3)	N2B—C7B—C8B—C13B	168.7 (3)
N2A—C7A—C8A—C9A	0.0 (4)	N1B—C7B—C8B—C9B	168.0 (3)
N1A—C7A—C8A—C13A	−1.2 (4)	N2B—C7B—C8B—C9B	−11.8 (4)
N2A—C7A—C8A—C13A	179.3 (2)	C13B—C8B—C9B—C10B	0.4 (4)
C13A—C8A—C9A—C10A	−0.7 (4)	C7B—C8B—C9B—C10B	−179.2 (2)
C7A—C8A—C9A—C10A	178.5 (3)	C8B—C9B—C10B—C11B	−0.9 (4)
C8A—C9A—C10A—C11A	0.9 (4)	C9B—C10B—C11B—C12B	−0.4 (4)
C9A—C10A—C11A—C12A	−0.2 (4)	C14B—O2B—C12B—C11B	4.6 (5)
C10A—C11A—C12A—O2A	179.4 (3)	C14B—O2B—C12B—C13B	−175.3 (3)
C10A—C11A—C12A—C13A	−0.6 (4)	C10B—C11B—C12B—O2B	−177.9 (3)
C14A—O2A—C12A—C11A	−6.6 (4)	C10B—C11B—C12B—C13B	2.1 (4)
C14A—O2A—C12A—C13A	173.4 (2)	C9B—C8B—C13B—O1B	−178.5 (3)
C11A—C12A—C13A—O1A	−179.0 (2)	C7B—C8B—C13B—O1B	1.1 (4)
O2A—C12A—C13A—O1A	1.0 (3)	C9B—C8B—C13B—C12B	1.3 (4)
C11A—C12A—C13A—C8A	0.7 (4)	C7B—C8B—C13B—C12B	−179.1 (3)
O2A—C12A—C13A—C8A	−179.3 (2)	O2B—C12B—C13B—O1B	−2.8 (4)
C9A—C8A—C13A—O1A	179.7 (2)	C11B—C12B—C13B—O1B	177.3 (3)
C7A—C8A—C13A—O1A	0.4 (4)	O2B—C12B—C13B—C8B	177.4 (3)
C9A—C8A—C13A—C12A	0.0 (4)	C11B—C12B—C13B—C8B	−2.5 (4)
C7A—C8A—C13A—C12A	−179.4 (2)	C12B—O2B—C14B—C15C	−87.0 (5)
C12A—O2A—C14A—C15A	−177.4 (2)	C12B—O2B—C14B—C15B	179.2 (4)
C7B—N1B—C1B—C2B	178.6 (3)		

### Hydrogen-bond geometry (Å, °)

**Table:**

*D*—H···*A*	*D*—H	H···*A*	*D*···*A*	*D*—H···*A*
O1A—H1OA···N1A	0.91 (4)	1.67 (4)	2.557 (4)	164 (4)
N2A—H1NA···O1B	0.81 (4)	2.14 (4)	2.865 (3)	149 (3)
N2A—H1NA···O2B	0.81 (4)	2.57 (4)	3.199 (3)	136 (3)
O1B—H1OB···N1B	0.97 (4)	1.67 (4)	2.567 (3)	151 (3)
N2B—H1NB···O1A^i^	0.94 (4)	1.95 (4)	2.877 (3)	167 (4)
N2B—H1NB···O2A^i^	0.94 (4)	2.55 (4)	3.136 (3)	121 (3)
C4A—H4AA···Cg1^ii^	0.93	2.80	3.590 (4)	143
C14B—H14C···Cg2^iii^	0.97	2.84	3.721 (5)	152
C15B—H15D···Cg3^iv^	0.96	2.76	3.715 (8)	176

Symmetry codes: (i) −*x*, *y*+1, −*z*+1/2; (ii) *x*, −*y*−1, *z*−1/2; (iii) −*x*, *y*, −*z*+1/2; (iv) −*x*+1/2, −*y*+1/2, −*z*+1.
